# A Medium-Throughput Single Cell CRISPR-Cas9 Assay to Assess Gene Essentiality

**DOI:** 10.1186/s12575-015-0028-4

**Published:** 2015-11-14

**Authors:** A. R. Grassian, T. M. E. Scales, S. K. Knutson, K. W. Kuntz, N. J. McCarthy, C. E. Lowe, J. D. Moore, R. A. Copeland, H. Keilhack, J. J. Smith, J. A. Wickenden, S. Ribich

**Affiliations:** Epizyme, Inc., 400 Technology Square, 4th Floor, Cambridge, MA 02139 USA; Horizon Discovery Ltd, 7100 Cambridge Research Park, Waterbeach, Cambridge, CB25 9TL UK

**Keywords:** CRISPR, Target validation, EZH2, Target identification, Epigenetics

## Abstract

**Background:**

Target selection for oncology is a crucial step in the successful development of therapeutics. Clustered regularly interspaced short palindromic repeats (CRISPR)-Cas9 editing of specific loci offers an alternative method to RNA interference and small molecule inhibitors for determining whether a cell line is dependent on a specific gene product for proliferation or survival. In our initial studies using CRISPR-Cas9 to verify the dependence on EZH2 activity for proliferation of a SMARCB1/SNF5/INI1 mutant malignant rhabdoid tumor (MRT) cell line, we noted that the initial reduction in proliferation was lost over time. We hypothesized that in the few cells that retain proliferative capacity, at least one allele of EZH2 remains functional. To verify this, we developed an assay to analyze 10s-100s of clonal cell populations for target gene disruption using restriction digest and fluorescent fragment length analyses.

**Results:**

Our results clearly show that in cell lines in which EZH2 is essential for proliferation, at least one potentially functional allele of EZH2 is retained in the clones that survive.

**Conclusion:**

This assay clearly indicates whether or not a specific gene is essential for survival and/or proliferation in a given cell line. Such data can aid the development of more robust therapeutics by increasing confidence in target selection.

**Electronic supplementary material:**

The online version of this article (doi:10.1186/s12575-015-0028-4) contains supplementary material, which is available to authorized users.

## Background

Despite large scale genomic sequencing efforts [1, 2], translation of the resulting data into successful treatment in the clinic has been difficult. Furthermore, many targets reported in the literature to be drivers of cancer have not been confirmed on further analysis [3, 4]. The use of RNA interference (RNAi) for target validation allows the effect of gene knockdown on proliferation to be rapidly assessed ; however, these reagents might not always lead to sufficient mRNA knockdown to evoke a phenotypic effect. For example, an RNAi screen to identify epigenetic dependencies found SMARCA2 as a potential target in *SMARCA4*-mutant cell lines [5]. Yet the screen failed to identify EZH2 in a *SMARCB1* mutant MRT cell line, G-401 [5], despite the fact that small-molecule-mediated inhibition of EZH2 in this cell line can induce cell death [6, 7]. Similarly, two independent pooled shRNA screens in primary cells from mouse models of MLL–AF9 acute myeloid leukemia (AML) did not identify DOT1L as an essential enzyme for these tumors [8, 9], even though *Dot1l* knockout mouse models [10, 11] and a small molecule DOT1L inhibitor [12] have indicated a requirement for DOT1L in MLL-driven AML. These data imply that for certain classes of targets, nearly complete inhibition of enzyme function is required to observe meaningful biological phenotypes.

The CRISPR (clustered regularly interspaced short palindromic repeats)–Cas9 system allows for a rapid, cost-effective and precise knockout of target genes through the introduction of insertions/deletions (indels) [13–16], and should therefore provide the necessary tools to observe phenotypes that require complete loss of protein function. To test this hypothesis, we carried out a proof-of-concept study using *EZH2* as a target for CRISPR–Cas9 mediated knockout in cell lines. EZH2 (Enhancer of Zeste 2) is the catalytic subunit of the polycomb repressive complex 2 (PRC2) and has histone methyltransferase activity that catalyzes the mono-, di- and tri-methylation of lysine 27 on histone 3 (H3K27) [17, 18]. Work using RNAi knockdown and small molecule inhibitors of EZH2 has shown that EZH2 is required for the proliferation of *EZH2* mutant lymphoma cells, *SMARCB1*-deficient MRT cells and cells from other cancer types [6, 7, 19–23].

As expected, the disruption of *EZH2* by CRISPR–Cas9 at early time points led to a decrease in the proliferation rate of an EZH2-dependent cell line, but not an EZH2-independent cell line. However, after 2 weeks the remaining cells in the EZH2-dependent cell line expressed near wild-type levels of EZH2 and showed comparable proliferation rates to a control cell line, whereas the EZH2-independent cell line maintained low levels of EZH2. This suggests that in the EZH2-dependent cell line, the cells in which CRISPR–Cas9 has successfully targeted *EZH2* rapidly arrest and/or are lost from the cell population, and that the cells that grow out after two weeks are those in which *EZH2* has not been effectively targeted and thus retain at least one functional copy of *EZH2*. If correct, this hypothesis indicates that carrying out CRISPR–Cas9 studies for individual target genes without analyzing single cell clones could produce misleading results. However, single cell cloning can be time-consuming and a large number of clonal cell populations need to be analyzed to derive accurate conclusions owing to the heterogeneous genotypes that could arise. Therefore, we have developed an assay that allows for the rapid measurement of gene knockout rates in 10’s–100’s of single cell clones. This assay uses both restriction digest analysis and fluorescent undigested fragment length analysis to assess the disruption status of each clone, with the assumption that in a cell line for which a gene is required, very few (if any) clones will emerge in which the gene of interest is homozygously knocked out. Using this protocol we were able to quickly and accurately assess EZH2 essentiality in four cell lines, proving that the assay is an accurate, quick and robust measure of gene essentiality. Importantly, this approach should be applicable to any target gene of interest that is expected to affect cell proliferation or survival, thus providing a crucial insight into gene essentiality and target identification.

## Results

### The Anti-Proliferative Effect of EZH2 Knockout does not Persist in an EZH2-Dependent Cell Population

**Fig. 1:**
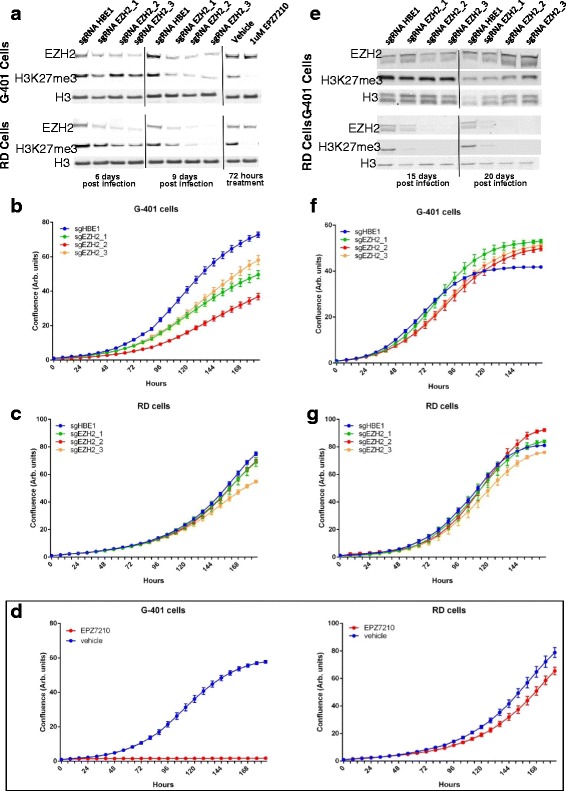
The anti-proliferative effect of EZH2 knockout does not persist in an EZH2-dependent cell population. G-401 and RD cells were infected with a lentiviral vector encoding Cas9 and each of three sgRNAs targeting *EZH2*. **a** The western blots show that 6 and 9 days after infection, EZH2 protein levels are decreased in both the G-401 cells and RD cells compared with a control sgRNA targeting the hemoglobin 1 (*HBE1*) gene. H3K27me3 levels are also decreased, whereas H3 levels are unchanged. The right hand panel indicates that treatment of G-401 cells and RD cells with a small molecule inhibitor of EZH2 for 72 h also decreases H3K27me3 levels, but has no effect on the expression levels of EZH2 and H3 as expected [6]. **b**, **c** Proliferation rate of cells from panel **a**. All 3 sgRNAs targeting EZH2 resulted in reduced rates of proliferation in G-401 cells and had little effect on the proliferation rate of the RD cells. **d** Proliferation rate of cells from panel **a** treated with or without a small molecule inhibitor of EZH2. **e** Western blot data showing that at 15 and 20 days post-infection, EZH2 expression levels are at near control levels in the G-401 cells infected with one of three independent sgRNAs targeting *EZH2*. A recovery in the levels of H3K27me3 was also evident. At the same time points, EZH2 protein expression remained low in RD cells infected with the same sgRNAs and H3K27me3 levels remained undetectable. **f**, **g** Proliferation rate of cells from panel **e**. The decrease in proliferation rates induced by *EZH2* knockout was no longer evident at 20 days post-infection in the G-401 cells, while proliferation rates remain unaltered in RD cells expressing one of three independent sgRNAs targeting EZH2. All data in this figure are representative of at least two independent experiments. Error bars show standard error mean for six technical replicates

We and others have previously found that *SMARCB1*-deficient but not *SMARCB1*-wild type MRT cell lines are sensitive to inhibition of EZH2 [6, 7, 23]. The CRISPR–Cas9 system allows for precise knockout of target genes through the introduction of insertions/deletions (indels), and provides the necessary tools to observe phenotypes that require complete loss of protein [13–16]. To test this approach, we carried out a proof-of-concept study using *EZH2* as a target for knockout in *SMARCB1*-negative (G-401) MRT cell line and *SMARCB1*-wild type (RD) rhabdomyosarcoma cell line; the former but not latter are sensitive to inhibition of EZH2 [6, 7, 23]. As expected, CRISPR–Cas9 targeting of *EZH2* led to a decrease in EZH2 protein and levels of H3K27me3 in both the G-401 and RD cells at 6 and 9 days post infection (Fig. 1a). Knockout of *EZH2* in G-401 cells also resulted in a decreased proliferation rate while the proliferation rate of RD cells was not affected (Fig. 1b and c). These results are concordant with our previously reported data using an EZH2 inhibitor [6], and confirmed here with EPZ007210, another highly selective and potent inhibitor of EZH2 activity [24] (Fig. 1a and d and Additional file 1: Figure S1). Thus, at an early time point following CRISPR–Cas9 gene editing, the phenotypic outcome mirrors that of small molecule inhibition in these cell lines.

Given the dependence of G-401 cells on EZH2 activity, we investigated how cells behaved at later time points after *EZH2* knockout. Surprisingly, EZH2 expression had already been restored to near control levels in the G-401 but not RD cells by 15 or 21 days post-infection (Fig. 1e), and the proliferation rate in the G-401 cells was restored to approximately control levels (Fig. 1f and g). As knockout results in a permanent and heritable loss of *EZH2*, expression cannot be recovered post knockout. Thus, these data suggest that the cells remaining at later time points likely retain at least one functional copy of *EZH2*. If this hypothesis is correct, the surviving G-401 cells will either retain a wild-type allele of *EZH2* or will have in-frame indels that do not affect EZH2 protein function. The DNA breaks made by Cas9 are repaired by the imprecise non-homologous end joining DNA repair pathway and some of these mutations could be in-frame indels that retain function, as has been seen for other genes [14, 25–27]. These results illustrate the potency of CRISPR–Cas9 for the generation of gene knockouts and also uncover some challenges with using CRISPR–Cas9 to assess the consequences of the deletion of an essential gene for target identification and validation.

### Assay Work Flow Summary for Single Cell CRISPR–Cas9 Essentiality Assay

Based on the above data, we set out to design and test an assay that would more rapidly and accurately allow the use of CRISPR–Cas9 reagents to test the essentiality of a single gene. After CRISPR–Cas9 targeting, our assay is designed to examine 1) whether the cells that continue to proliferate in a target-dependent cell line are those that retain at least one in-frame allele (either edited or unedited) and 2) whether cells that continue to proliferate in target-independent cell line have alleles that are mostly edited and have out-of-frame alterations. A common technique to study gene knockout phenotypes is to examine single cell clones and we hypothesized that for a cell line which is dependent on a certain gene, it should be difficult, if not impossible, to generate clonal homozygous knockouts for the gene of interest. Conversely, in cell lines for which a large number of homozygous knockout single cell clones are recovered, it would be unlikely that the growth of that cell line is dependent on the gene of interest. Therefore, we developed a protocol that allowed us to examine gene knockout rates in 10’s to 100’s of single cell clones in a medium-throughput manner. This protocol also assesses whether in-frame or out-of-frame alleles for the gene of interest are produced. Thus, this assay allows for the rapid assessment of gene essentiality.

**Fig. 2:**
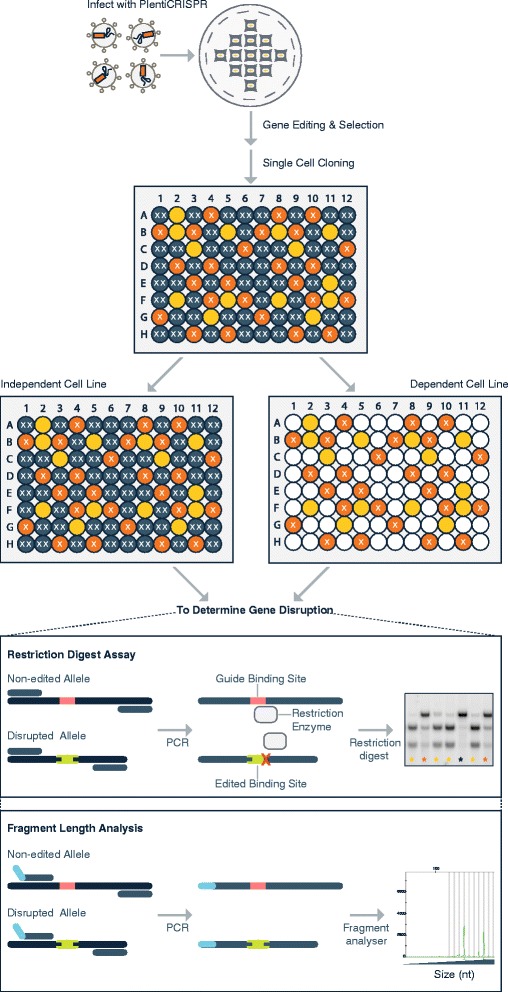
Assay work flow summary for single cell CRISPR-Cas9 essentiality assay. Cells are infected with pLentiCRISPR containing Cas9 and a validated sgRNA targeting a gene of interest. After virus infection, the cells are selected with puromycin for a minimum of 2 weeks before single cell dilution in 384-well plates. Blue wells labeled with two “XX” indicate cells with homozygous disrupted alleles, orange with “X” indicate cells with heterozygous disrupted alleles, and yellow indicate cells in which the target alleles are unedited. Two medium-throughput methods are used to examine the zygosity of the gene of interest in each of the isolated clones. For the restriction digest, the region around the sgRNA target site is amplified by PCR and subject to restriction digest. PCR primers are chosen such that DNA fragments of unequal length are produced if the restriction site remains intact. Alleles that have been edited should have the restriction site disrupted (represented by the green band) and therefore will not be cut by the restriction enzyme. Unedited alleles (represented by the pink band) should retain an intact restriction site and should be cut by the restriction enzyme. The gel shows examples of clones in which both alleles have been edited (*blue asterisk*), one allele has been edited (*orange asterisk*) and no alleles have been edited (*yellow asterisk*). Fluorescent fragment length analyses are also used to assess indel status. PCR primers that incorporate a fluorescent tag are used to amplify an area around the sgRNA binding sequence and the Cas9 cut site (indicated by the pink band). A fragment of un-edited length suggests a lack of disruption, whereas a fragment that is either longer or shorter than the un-edited length suggests the presence of an indel (indicated by the green band)

The work-flow for this assay is shown in Fig. 2. In brief, a sgRNA is chosen that has a Cas9 cleavage site that overlaps with a restriction digest site, thus allowing for rapid analysis for the presence of indels (as described below). After virus infection, the cells are transduced with lentivirus containing Cas9 and the sgRNA, and selected with a relevant antibiotic for at least 2 weeks before single cell dilution. This prolonged antibiotic selection ensures that the majority of the target alleles will have been edited, and that colonies derived from the single cells will be uniform. Wells with a single cell are marked and colony growth is monitored until sufficient cell numbers are present in the colony to enable assessment of the knockout status by PCR-based techniques. Two medium-throughput methods are then used to examine the zygosity of the gene of interest in each of the isolated clones: restriction digest and fluorescent undigested fragment length analysis. For the restriction digest, PCR primers are chosen such that DNA fragments of unequal length are produced if the restriction site remains intact (as illustrated in Fig. 2). Although the restriction digest assay can provide an estimate for indel frequency, it does not correctly identify indels that do not result in alteration of the restriction digest site, or for indels that abolish the site but do not result in mis-sense or non-sense transcripts (e.g. in-frame indels or single base alterations). Thus, fluorescent fragment length analysis of the undigested PCR product is also used to assess indel status. A fragment of wild-type length suggests either a lack of disruption or in-frame nucleotide alteration(s), whereas a fragment that is not wild-type length indicates the presence of indels. By combining these techniques, the assessment of zygosity can be evaluated in a medium-throughput manner in single cell clones and the functional status of the gene of interest inferred. Importantly, this technique should be applicable to any gene, with the only requirements being the identification of a functional sgRNA for the target gene and cell lines that can form single cell clones.

### Proof of Concept for Medium-Throughput Assay with EZH2

Four cell lines were used as a proof-of-concept for this assay: EZH2 inhibitor insensitive RD cells (*SMARCB1* wild-type), and the SMARCB1-negative cell lines G-401, G-402 and KYM-1 which are dependent on EZH2 catalytic activity for proliferation [6]. Having verified that these cell lines could generate colonies from single cells at a sufficient rate (Additional file 1: Table S1), each line was infected with an EZH2 sgRNA and Cas9; this sgRNA substantially reduces EZH2 expression and function (Fig. 1a; EZH2 sgRNA #3) with limited predicted off-target cut sites (data not shown) and contains a restriction digest site that spans the Cas9 cut site (Additional file 1: Figure S2). Cells were grown in the presence of puromycin for 17 days (by which time we anticipate that gene editing would have proceeded to completion at the *EZH2* locus), followed by single cell dilution.

**Fig. 3:**
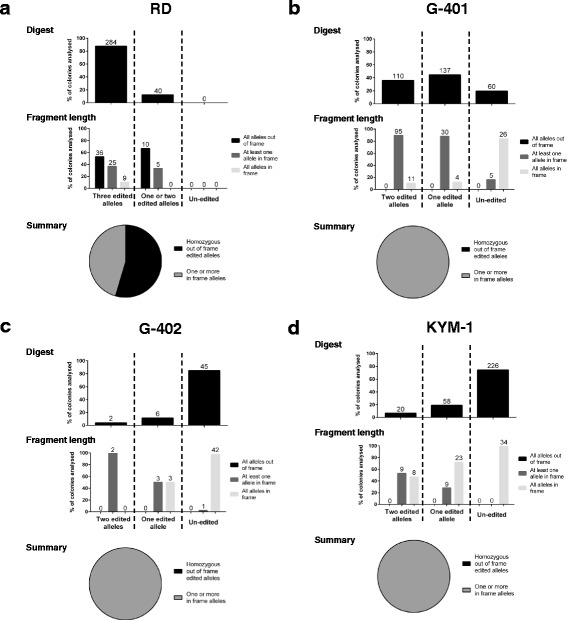
Restriction digest data qualified by fragment length analyses. **a**-**d** Collated percentages of homozygous edited, heterozygous and homozygous un-edited clones in each cell line. For each cell line, the top graph shows the restriction digest data and the bottom graph shows the fragment length analysis data. The actual numbers of clones analyzed are shown on top of each bar. The pie chart at the bottom of each cell line panel indicates the zygosity of the clones analyzed by the combined approaches of restriction digest and PCR fragment length analyses. Note that RD cells have 3 copies of *EZH2*, whereas G-401, G-402 and KYM-1 cells have two copies

We analyzed the colonies by restriction digest to assess the allelic status of *EZH2* (each of the cell lines examined has two copies of *EZH2*, with the exception of the RD cells which contain three copies). For the RD cells (EZH2-independent), the majority of the clones showed homozygous disruption at the *EZH2* locus (Fig. 3a and Additional file 1: Figure S3). However, for the three EZH2-dependent cell lines, the majority of the clones recovered had either unedited *EZH2* alleles or were heterozygous at this locus (one edited and one un-edited allele) as assessed by restriction digest analysis (Fig. 3b, c and d and Additional file 1: Figure S3). This differential outgrowth suggests that in the EZH2-dependent cell lines, at least one allele remains that is likely to be functional.

Although restriction digest analysis is an efficient technique to analyze editing frequency, the restriction digest analysis does not offer complete information about the nature of the indels present at the *EZH2* locus. For example, in-frame indels or single base alterations could appear as disruptions by restriction digest, but might not affect protein function. To examine this further, we used fluorescent fragment length analysis on a subset of the clones that had been analyzed by restriction digest. In the RD cells, all of the clones had at least one of the three *EZH2* alleles edited (a fragment length smaller or greater than 394 bp), and in over half of the clones, all three alleles had been edited resulting in out-of-frame indels (Fig. 3a and Additional file 1: Figure S4). Conversely, in the EZH2-dependent cell lines, almost all clones had at least one fragment length that was 394 bp, indicating the presence of un-edited or in-frame alterations (Fig. 3b, c and d and Additional file 1: Figure S4). The rare clones in the EZH2-dependent cells that seemed to have no PCR products of wild-type length were those in which the fragment lengths scored just below 394 bp or just above 394 bp (Additional file 1: Figure S4), which could be caused by fragment length analysis assay imprecision.

To understand better the reason why some clones appear as homozygous disrupted in the digest assay but heterozygous disrupted in the fragment length assay, we sequenced the relevant PCR products. Four examples are shown in Table 1. In both of the examples from the G-401 cells, one allele of *EZH2* is disrupted by a deletion (16 bp and 47 bp, respectively) and the other allele in both clones has a single nucleotide change that disrupts the Fau1 site. Both alleles have been edited and the restriction digest in these clones correctly indicated a homozygous disruption, and the fragment length PCR also indicated correctly the presence of an allele of undisrupted length. The sequencing confirmed that these clones have retained an in-frame *EZH2* allele. In the two KYM-1 clones, both alleles have also been edited. Again, the restriction digest data indicated homozygous edited alleles for both clones. For clone C, the fragment length analysis indicated one edited allele and one allele of 394 bp. In this case the sequencing data indicated an in-frame 3 bp alteration. Clone D from KYM-1 cells showed both fragment lengths corresponded with an un-edited fragment length, and this was confirmed by the sequence data, which showed a single nucleotide alteration in both alleles; this mutation disrupts the FauI site, but retains the reading frame, suggesting that EZH2 remains expressed (and likely functional) in these clones. The sequencing data also allowed us to be sure that the detection of a PCR product that was just above or just below 394 bp represented alleles in which single in-frame nucleotide changes had occurred: this is reflected in the summary graph and pie charts in Fig. 3, where all clones from EZH2 dependent cell lines were verified as having at least one allele of 394 bp. Interestingly, the sequencing data combined with the fragment length analyses (Fig. 3, Table 1 and Additional file 1: Figure S4) also indicate that some of the clones from the EZH2-dependent cell lines might have originated from the same precursor cells, further indicating that the clones that grow out in EZH2-dependent cell lines represent a small proportion of the original cell population.

**Table 1:**
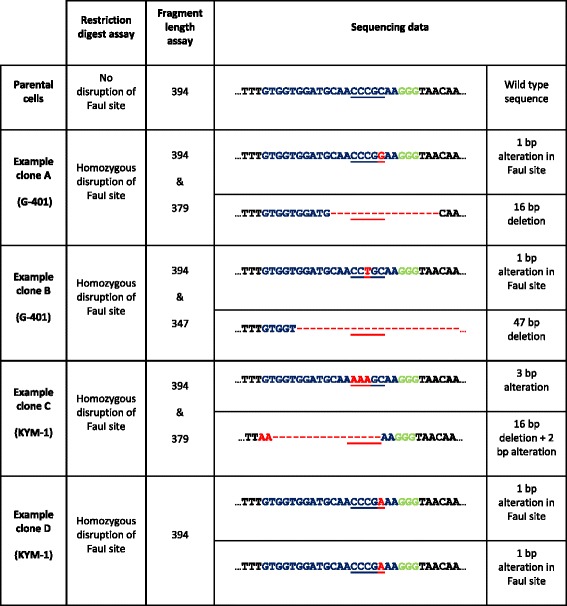
Sequencing allows further refinement of zygosity

Sequence analysis of specific clones to verify the status of *EZH2* alleles. In both of the examples from the G-401 cell clones (**A** and **B**), one allele of *EZH2* is disrupted by a deletion (16 bp in clone A and 47 bp in clone B) and the other allele in both clones has a 1 bp mutation that disrupts the Fau1 site. Both alleles have been edited and the restriction digest in these clones correctly indicated a homozygous disruption: the fragment length PCR analysis also indicated correctly the presence of an allele of undisrupted length. The sequencing data shows that in all probability these clones have retained a functional *EZH2* allele owing to an in frame mutation. In both of the KYM-1 clones (**C** and **D**), both alleles have been edited. Again, the digest data correctly indicated homozygous edited alleles for both clones. For clone **C**, the fragment length analysis indicated heterozygous edited alleles which was confirmed by the sequencing data showing one allele with an in frame 3pb alteration. Clone **D** from KYM-1 cells showed both fragment lengths corresponded with an un-edited fragment length, and this was confirmed by the sequence data, which showed a 1 bp alteration in both alleles. This explains why the restriction digest indicated homozygous edited alleles because the restriction digest site is mutated in both cases. These data also suggest that EZH2 remains expressed in these clones

Thus, the combined analyses of the restriction digest data and the fragment length data help to clearly define the zygosity of the clonal cells, allowing for confirmation of EZH2 dependent and non-dependent cell lines. Sequencing analysis can also be used to indicate the presence or absence of an in-frame allele.

## Discussion

CRISPR–Cas9 editing of specific loci offers an alternative method to RNAi and small molecule inhibitors for determining whether or not a cell line is dependent on a specific gene product for proliferation and/or survival. Our initial data indicated that knockout of *EZH2* using CRIPSR–Cas9 in a cell line that is dependent on EZH2 function for proliferation resulted in an expected proliferation defect. However, growth of this cell population over a prolonged period indicated that this effect was transient, most likely owing to the outgrowth of cells in which EZH2 function remains intact. In order to test our hypothesis that the cells that grow out after 20 days are those in which *EZH2* has either not been targeted, or in which the indels lead to in-frame alterations, we developed a medium throughput assay to analyze 10s–100s of clones for gene essentiality.

The results from our gene essentiality assay clearly show that in cell lines that are EZH2-independent, proliferation rates are unaltered, and the majority of clones have *EZH2* alleles that have been edited such that EZH2 function is likely lost. Conversely, in all three dependent cell lines, fewer clones survive the gene knockout process. Of those that do, retention of an in-frame or wild-type copy of one allele is observed in every clone. Thus, the combined analyses of the restriction digest data and the fragment length data help to clearly define the zygosity of the clonal cells, allowing for confirmation of EZH2-dependent and non-dependent cell lines. Sequencing analysis can also be used to indicate the presence or absence of an in-frame allele.

Although this assay can be used to help identify genes that are essential for the growth of specific cancer cell lines, it can also be used to determine whether a gene of interest is likely to be a poor drug target owing to its essentiality in many different cell lines (i.e. broad essentiality would be indicative of potential safety issues in the clinic). Therefore, our gene essentiality assay is applicable to diseases other than cancer. There are specific considerations that need to be addressed when using this assay. First, the sgRNAs chosen for this assay need to be assessed before-hand, specifically showing that they lead to a substantial decrease in target expression and that there are no proliferation effects that are due to off-target effects. In our work with EZH2 we were able to choose a sgRNA that was validated in a cell line in which EZH2 is not required for proliferation. This might be challenging to address for genes of interest in which no target-independent cell line has been identified. There are several algorithms that allow potential off-target binding sites to be assessed for each potential sgRNA and as CRIPSR–Cas9 screens become more widely used, the data generated from these screens should prove useful for the identification of guides that do not have off-target proliferation effects. Second, some genes might not be required for cell line proliferation on tissue culture plastic, but are essential for growth in soft agar or other matrix conditions [28–30]. Such an effect would be missed in our current assay, but could be easily addressed by including single cell assays in alternative tissue culture conditions. Third, as it is currently configured, this assay cannot be used in cell lines that do not grow or survive as single cells, thus probably limiting its use for primary cells and patient samples. Finally, although this assay can identify indels induced by Cas9 editing, it does not test for enzymatic function of these mutations.

## Conclusions

These findings highlight an important caveat to the use of CRISPR–Cas9 for target identification and validation, and present a robust assay to overcome these challenges. We have developed a novel medium-throughput assay to analyze gene essentiality in cell lines that are amenable to single cell dilution cloning. This method should aid both the understanding of whether the protein from a target gene of interest needs to be completely absent for a phenotypic effect or whether reduced expression is sufficient to induce the phenotype of interest. Such data will support the development of more robust cancer therapeutics.

## Methods

### Vector Construction and Virus Production

sgRNA pRSGC1-U6-sg-CMV-Cas9-2A-Puro plasmid and lentivirus for Figs. 1 and 2 were purchased from Cellecta. The sequences for the sgRNA’s are as follows: EZH2 sgRNA #1 ACCGGGGAAGCAGGGACTGAAACG; EZH2 sgRNA #2 ACCGGGTCCCAATTAACCTAGCAA; EZH2 sgRNA #3 ACCGGTGGTGGATGCAACCCGCAA. For additional studies, sgRNA #3 sequence was cloned into pLentiCrispr v2 at DNA 2.0 [31]. HEK293T cells were transfected with the pLentiCrispr-sgEZH2 construct and Virapower packaging system using Lipofectamine 3000 (Life Technologies). Media was replaced with DMEM, 10 % FBS, 1 % BSA after 6 h and harvested 65 h later. Viral particles were concentrated using Lenti-X concentrator (Clontech).

### Immunoblotting

Cells were lysed in RIPA buffer containing 10 % SDS and cOmplete Protease Inhibitor Cocktail Tablets (Roche). Lysates were spun at 16,000 × g at 4 °C for 30 min and normalized for protein concentration. Lysates were separated using SDS-PAGE, and transfer/blotting was performed as described previously [32]. Blots were imaged by fluorescent imaging using the Odyssey infrared imaging system (LI-COR Biosciences; Millennium Science, Surrey Hills, Australia). The following antibodies were used: EZH2 (Cell Signaling #3147), H3K27Me3 (Cell Signaling #9733) and total H3 (Cell Signaling #3683).

### Incucyte Proliferation Assays

Cells were plated in triplicate in 96 well plates and confluency measurements were taken every 2 h using an Incucyte Kinetic Imaging System (Essen BioScience).

### Functional Titration and Spinfection of Cell Lines

Cells were spinfected by placing one million cells per well in four wells of a 12 well plate in media containing 8 μg/ml polybrene. Multiple concentrations of virus (0–40 μl) were added and the plate was centrifuged at 568 xg for 2 h at 37 °C. Virus containing media was then removed and replaced with 1 ml of media without polybrene prior to incubation at 37 °C. The following day, cells were trypsinized and the cell suspension was divided into two wells of a 6-well plate and a lethal dose of puromycin (0.4 μg/ml) was added to one well. Seventy-two hours after plating, cells were trypsinized and counted and the percent age of transduced cells was determined. Cells infected with the optimal volume of virus were maintained for 17 days in puromycin prior to plating for single cell colonies.

### Single Cell Dilution of Cell Lines

All four cell lines had been previously evaluated for their ability to robustly grow from single cells. On the day of plating, cells were trypsinized, counted, diluted to a density of 0.75 and 1.5 cell per well and plated in five 384-well plates at each density by Multidrop dispenser (Thermo Scientific). Plates were imaged using a Cell Metric™ within 6 h of seeding, at intervals of seven days and at the time of harvest. When colonies had reached sufficient size (at least 25 % well confluence), data from the Cell Metric™ was used to generate a pick list for each cell line. Colonies were trypsinized and consolidated from multiple plates into a single 384-well plate for each cell line using a Hamilton Microlab Star robotic system. Samples were immediately lysed in DirectPCR lysis reagent (Viagen).

### Restriction Digest Analysis of EZH2 Targeted Colonies

The region around the Cas9 cut site targeted by the sgRNA was amplified by PCR. Primers used were as follows; forward AGGCAAACCCTGAAGAACTG, reverse ATGGCTCTCTTGGCAAAAAT. PCR products were digested with the restriction enzyme FauI, which directly overlapped the Cas9 cut site for the sgRNA targeting *EZH2*. Digested PCR products were resolved by agarose gel electrophoresis and assessed for the genotype of disruption of the FauI cut site (heterozygous disrupted, homozygous disrupted or homozygous un-disrupted).

### Fragment Length Analysis

The region around the Cas9 cut site targeted by the sgRNA in either *EZH2* or the ROSA26 locus was amplified by PCR using primers of the same sequence as for the digest analysis, with the forward primer tagged with FAM. Resultant PCR products were purified using Diffinity RapidTips (Sigma) and diluted 1:50 in H_2_O. Capillary electrophoresis was performed on the samples (Source Biosciences) using Liz-600 size standards; fragment length was determined using Genemapper5 software (Applied Biosystems).

## Additional File

Additional file 1:
**Figure S1.** Data relating to EPZ007210. **Table S1.** Verification that the cell lines used in the assay can grow from single cells. **Figure S2.** Restriction assay details. **Figure S3.** G-401 clones **Figure S4.** Fluorescent PCR fragment length raw data. (PDF 1729 kb)

## Abbreviations

AML: Acute myeloid leukemia
CRISPR: Clustered regularly interspaced short palindromic repeats
EZH2: Enhancer of zeste 2
Indels: Insertions/deletions
MRT: Malignant rhabdoid tumor
PRC2: Polycomb repressive complex 2
RNAi: RNA interference
shRNA: short hairpin RNA
