# Supplementary material for: A Medium-Throughput Single Cell CRISPR-Cas9 Assay to Assess Gene Essentiality
Source: Biol Proced Online. 2015 Nov 14;17:15. doi: 10.1186/s12575-015-0028-4 (PMC4647477; doi:10.1186/s12575-015-0028-4)
Supplement: Additional file 1: — Figure S1. Data relating to EPZ007210. Table S1. Verification that the cell lines used in the assay can grow from single cells. Figure S2. Restriction assay details. Figure S3. G-401 clones Figure S4. Fluorescent PCR fragment length raw data. (PDF 1729 kb) [file 12575_2015_28_MOESM1_ESM.pdf]

Supplementary data

Supplementary Figure 1

A

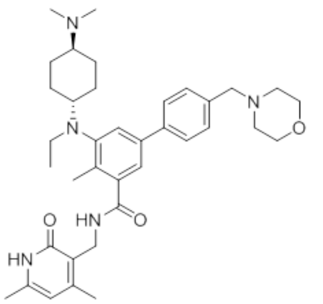

B

	EZH2 IC_{50} peptide v2 (μM)	EZH2 Y646F IC_{50} (μM)	WSU prolif IC_{50} (μM)	WSU ELISA IC_{50} (μM)
**EPZ007210**	0.00369	0.00454	0.07815	0.05978

(A) Structure of EPZ007210. Synthesis described in patent application WO2012142504 A1 (1). **(B)** Biochemical IC_{50} for wild-type EZH2 and EZH2 Y646F, and the IC_{50} for proliferation and H3K27 trimethylation in WSU-DLCL2 cells (1). **(C)** Representative examples for the data in panel B.

Supplementary Table 1

Verification that the cell lines used in the assay can grow from single cells

Cell line	% of wells containing single colonies
G-401	38%
G-402	13%
KYM-1	38%
RD	14%

Supplementary Figure 2

A

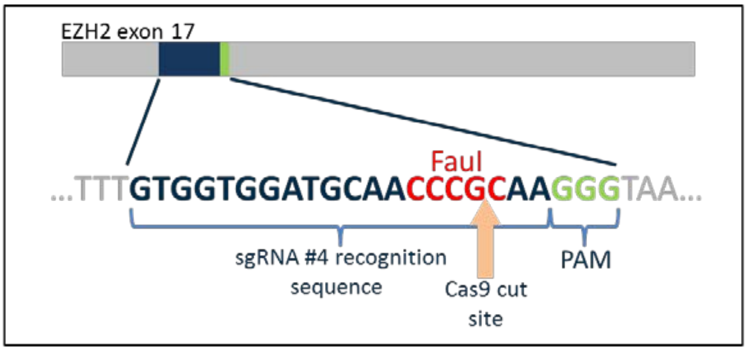

B

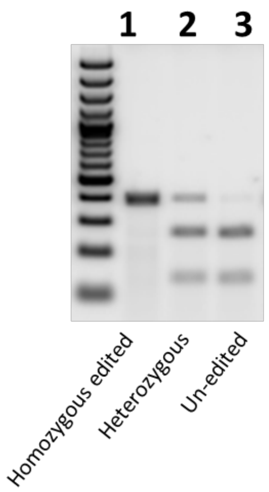

C

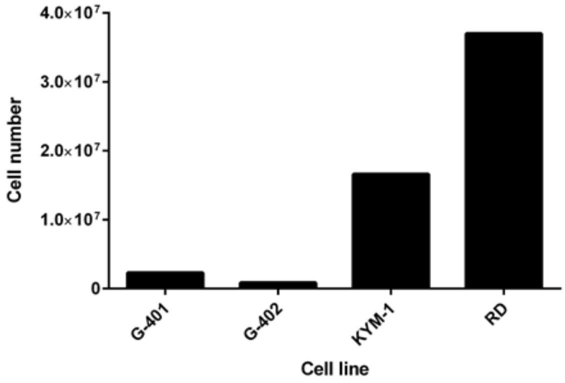

(A) sgRNA #3 binds to a DNA sequence that contains a restriction digest site for FauI that spans the Cas9 cut site. Alleles that have been edited should have the restriction site disrupted and therefore will not be cut by the restriction enzyme (example shown in lane 1 on the right hand panel). Unedited alleles should retain an intact restriction site and should be cut by the restriction enzyme (example shown in the right hand panel in lane 3). Clones with one edited and one un-edited allele have all 3 bands (example shown in lane 2, right hand panel). (**B**) The panels show representative restriction digest results Full restriction digest data for all clones analysed are shown in supplementary Figure 2 (n=1). (**C)** As expected, the EZH2 dependent cell lines proliferated less well after infection with pLentiCRISPR containing Cas9 and sgRNA#3 compared with RD cells (n=1).

Supplementary Figure 3

G-401 clones

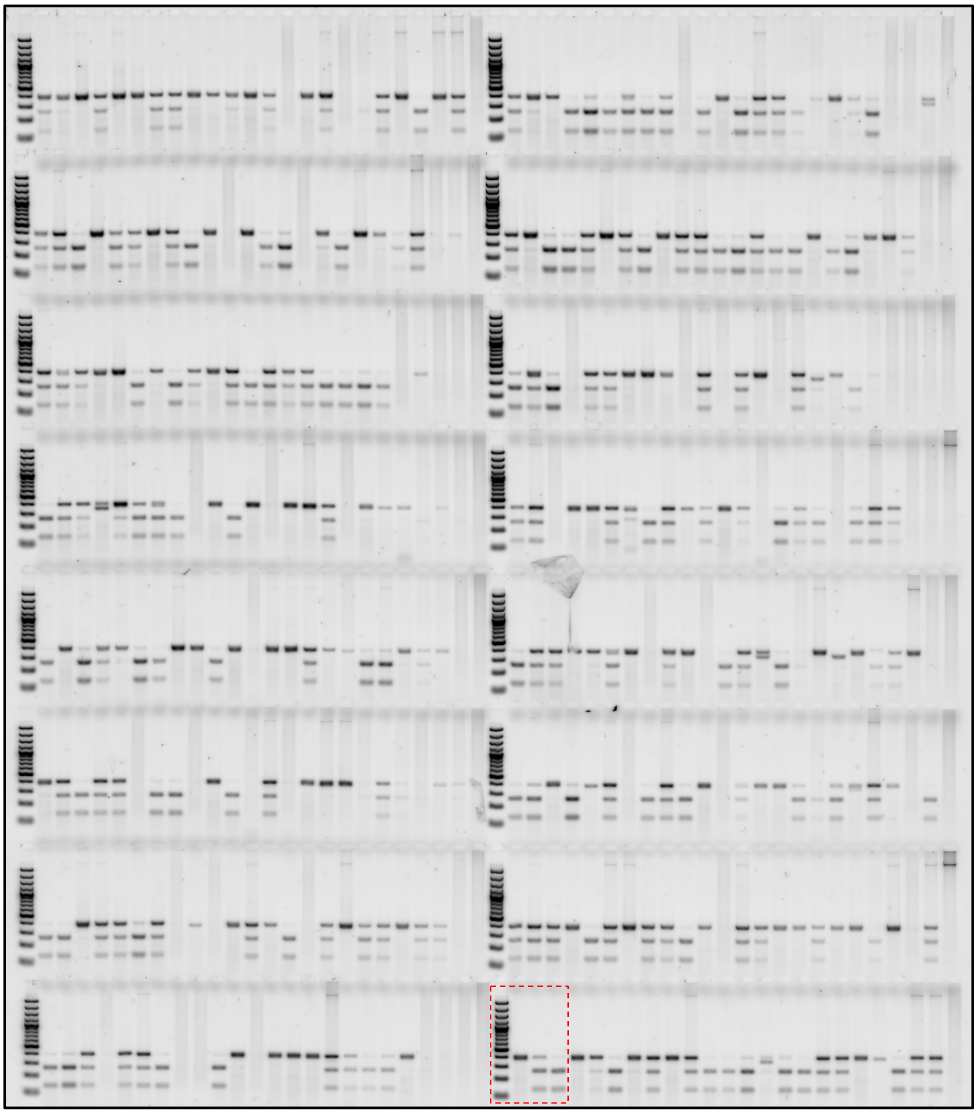

Note that many clones have one or both alleles edited in this cell line. Red dashed box indicates area of gel used in Supplementary Figure 2B.

G-402 clones

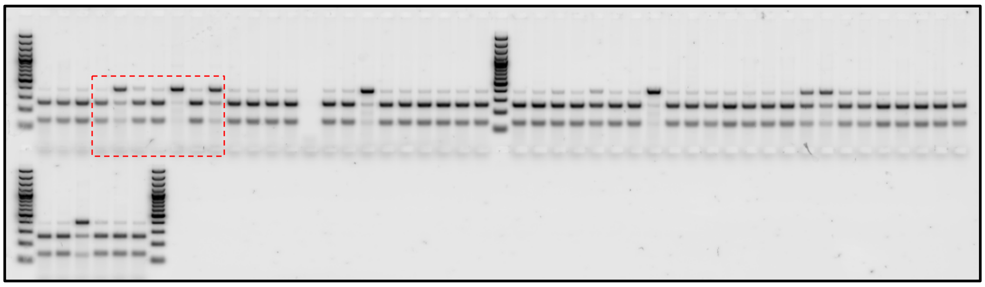

Only a few single cell clones grew in the G-402 cell lines compared with the other cell lines. The red dashed box indicates the region of the gel shown in Figure 2.

KYM-1 clones

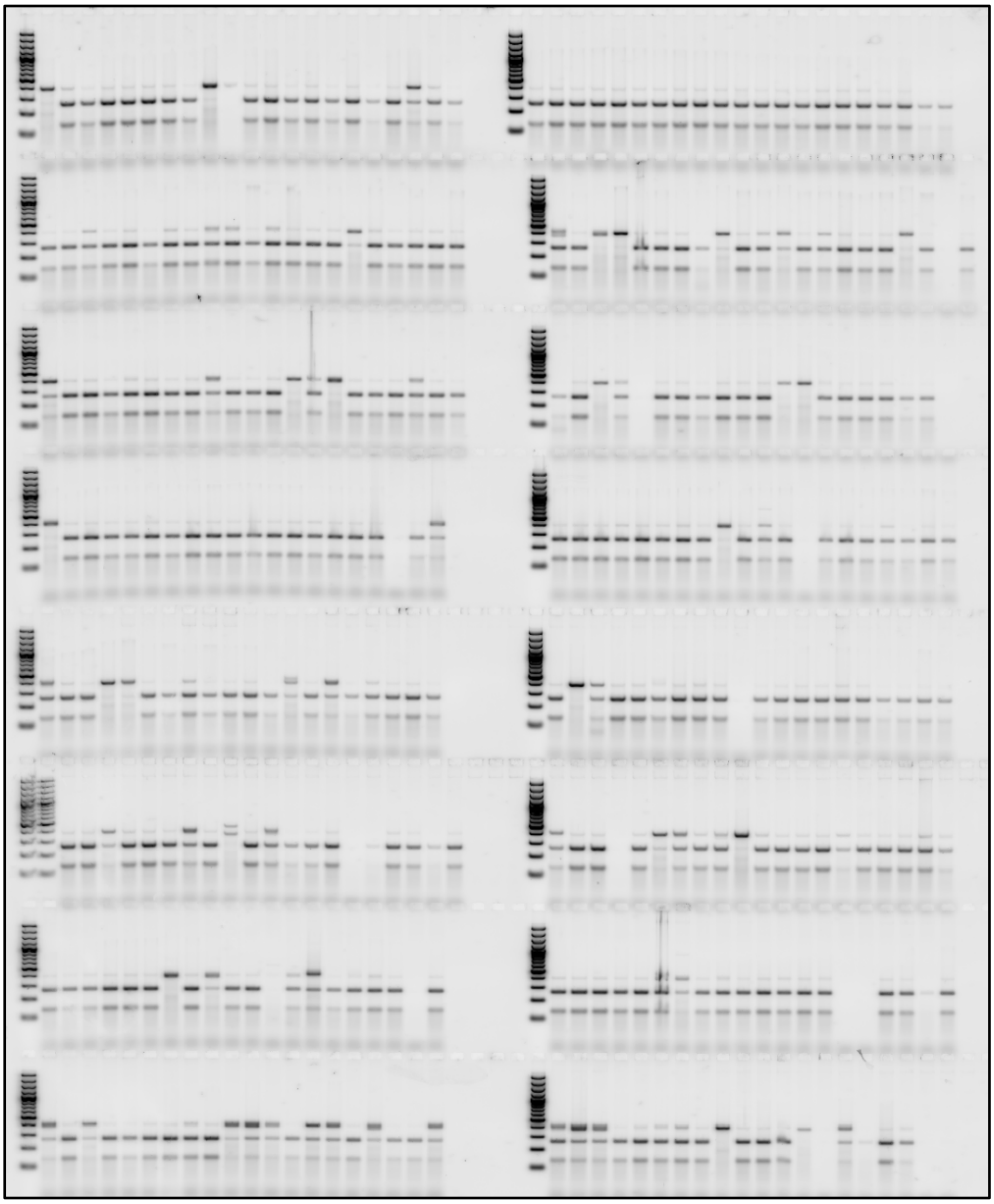

Note that clones with un-edited alleles predominate in this cell line.

RD clones

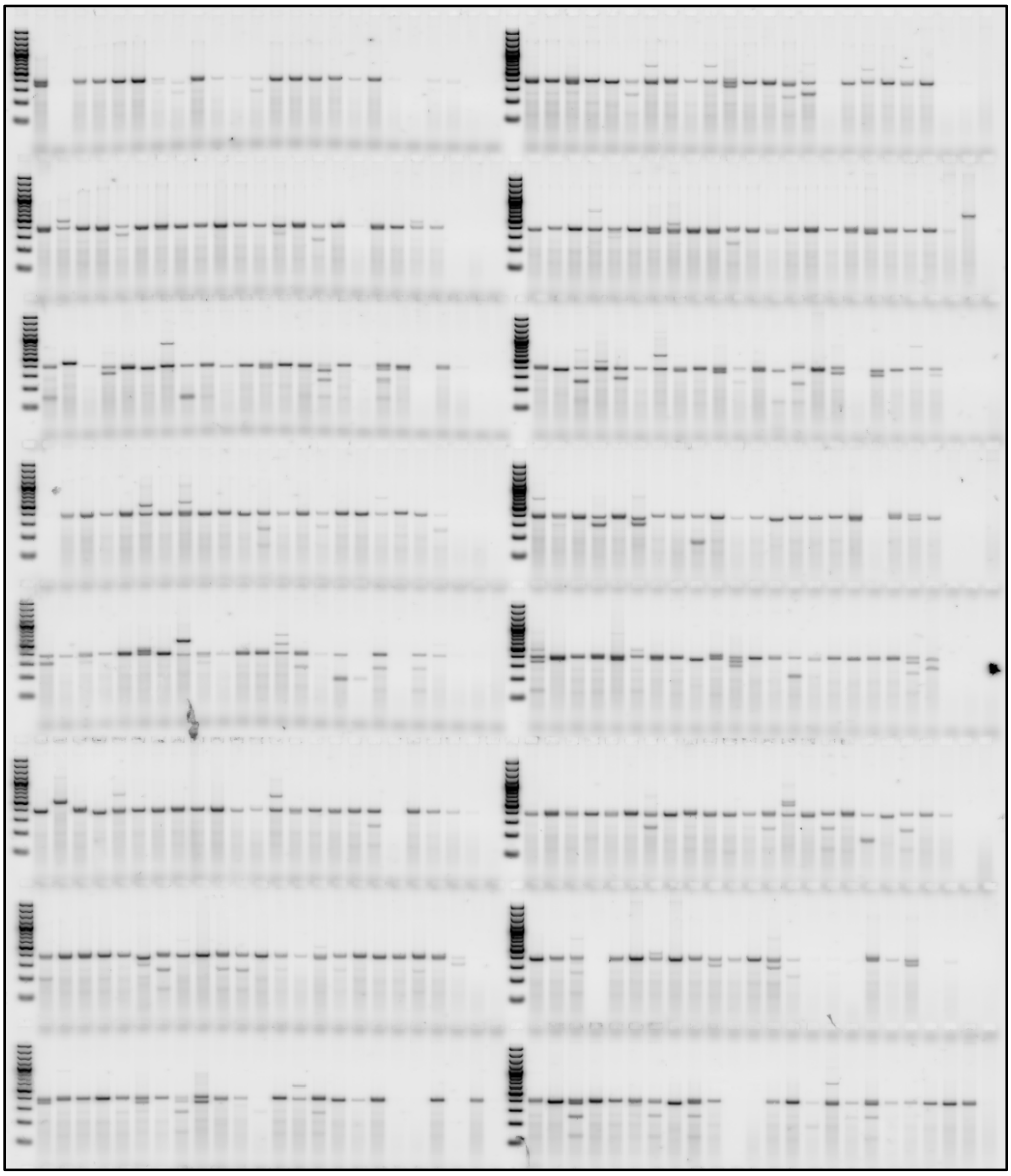

Note that all clones have edited alleles in this cell line; there are no clones where all alleles are un-edited.

Supplementary Figure 4

Fluorescent PCR fragment length raw data

These graphs show the fragment length data obtained for each clone analysed using this technique. The fragment lengths are coloured in accordance with the data obtained using the restriction digest assay.

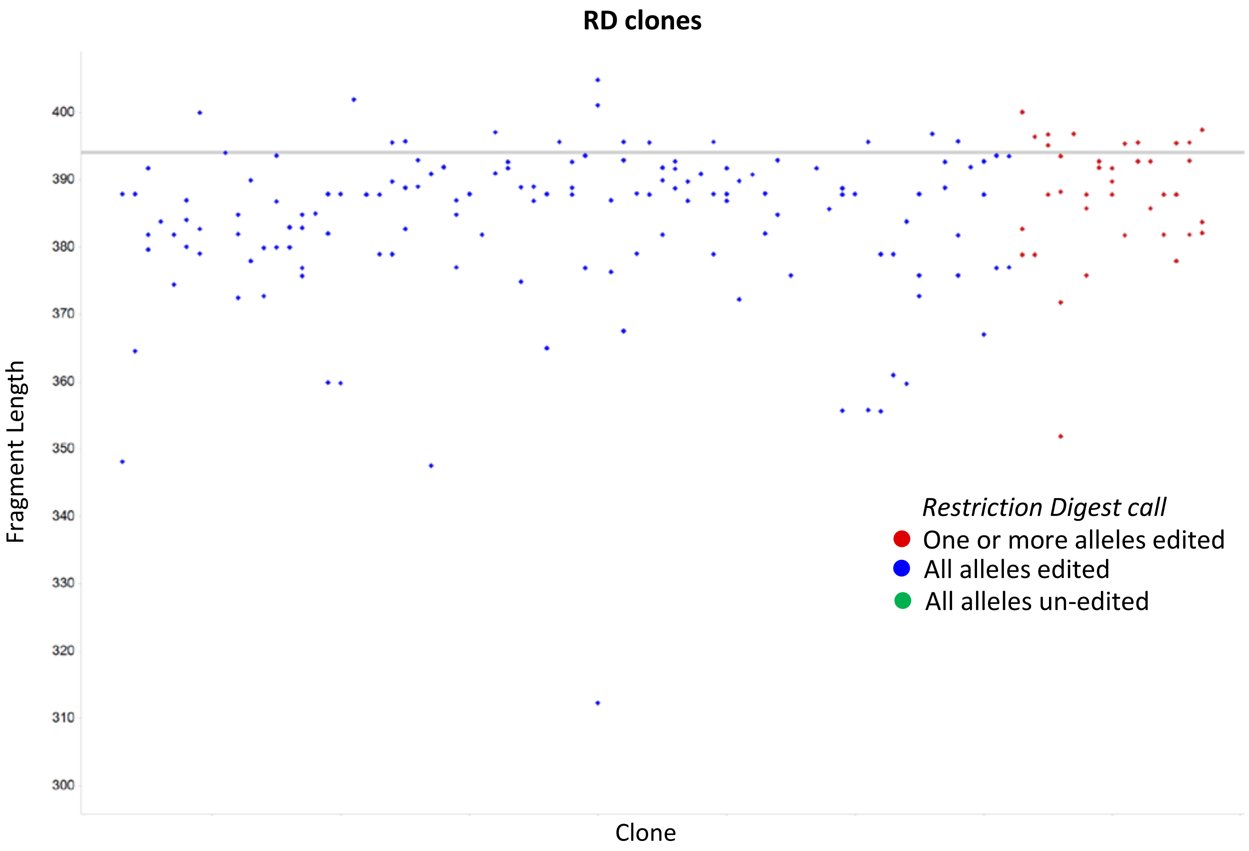

The digest data (supplementary Figure 2) indicated no-unedited alleles, and the fragment length data is in agreement. Note the varying lengths of the edited alleles, indicating that most of the clones analysed probably arose from individual edited cells in the initial population. The grey line represents the wild-type fragment length (394 bp).

G-401 clone

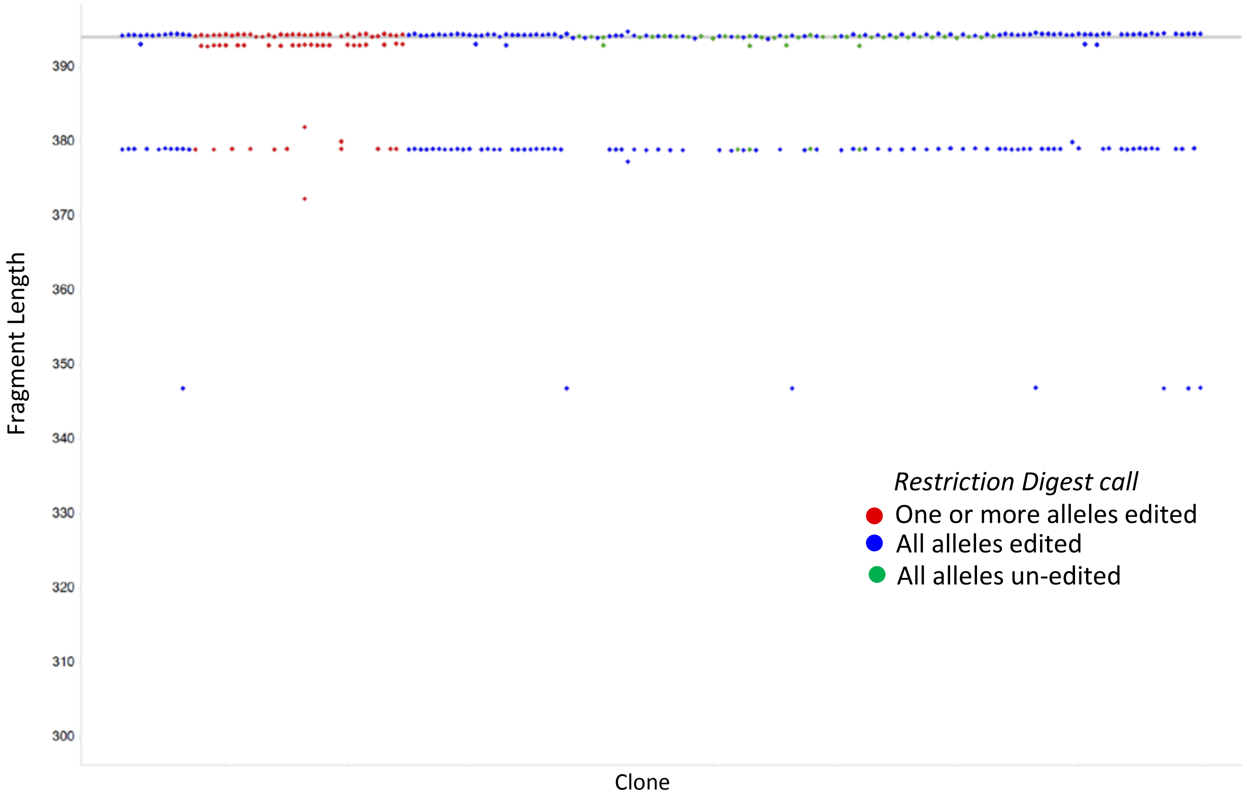

The common fragment lengths evident in these clones indicate that many of these clones are potentially sisters that arose from a small population of cells in which one allele of *EZH2* was initially edited. There are also a few clones in which there are 3 or more fragment length traces. Checking of the Cell Metric data indicate that these clones were not true 'singles' in that more than one cell was seeded in that particular well during the single cell cloning step. The grey line represents the wild-type fragment length (394 bp).

G-402 clone

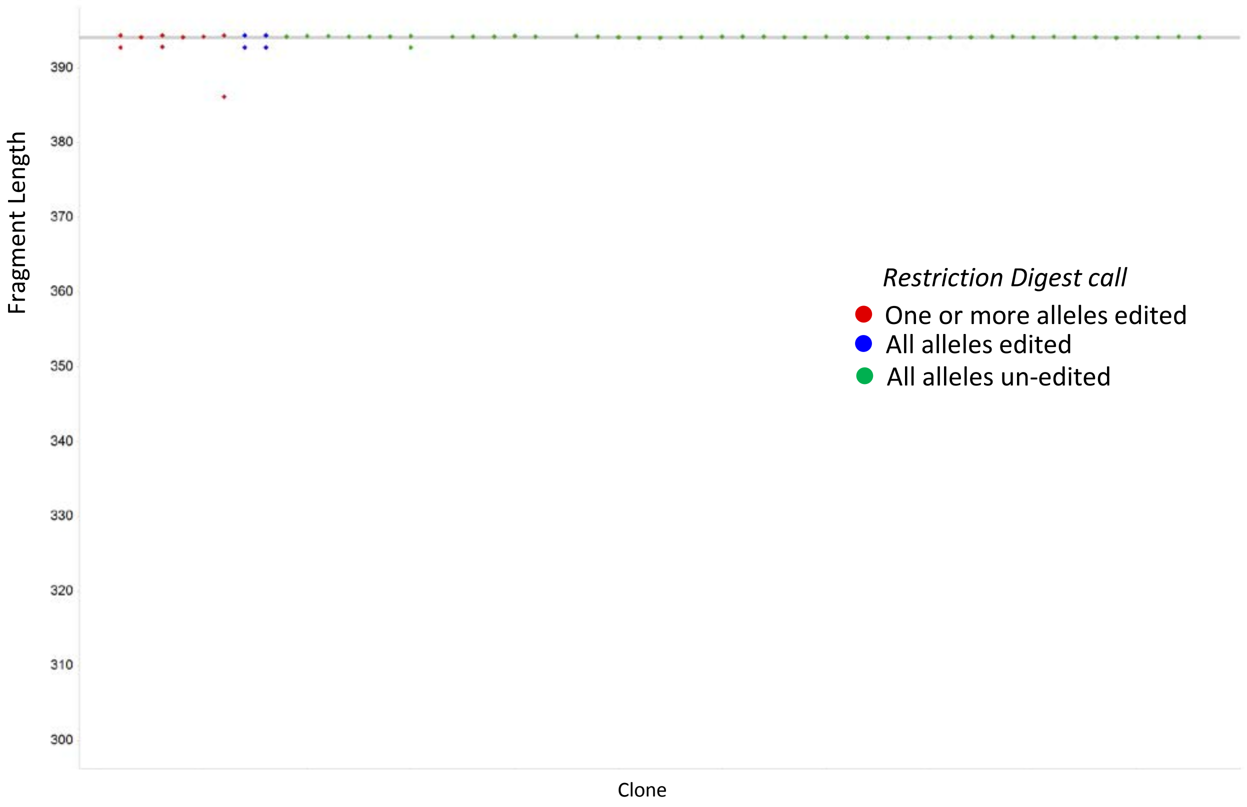

For most of the clones from this cell line only one fragment length was detected (394bp). The grey line represents the wild-type fragment length (394 bp).

KYM-1 clones

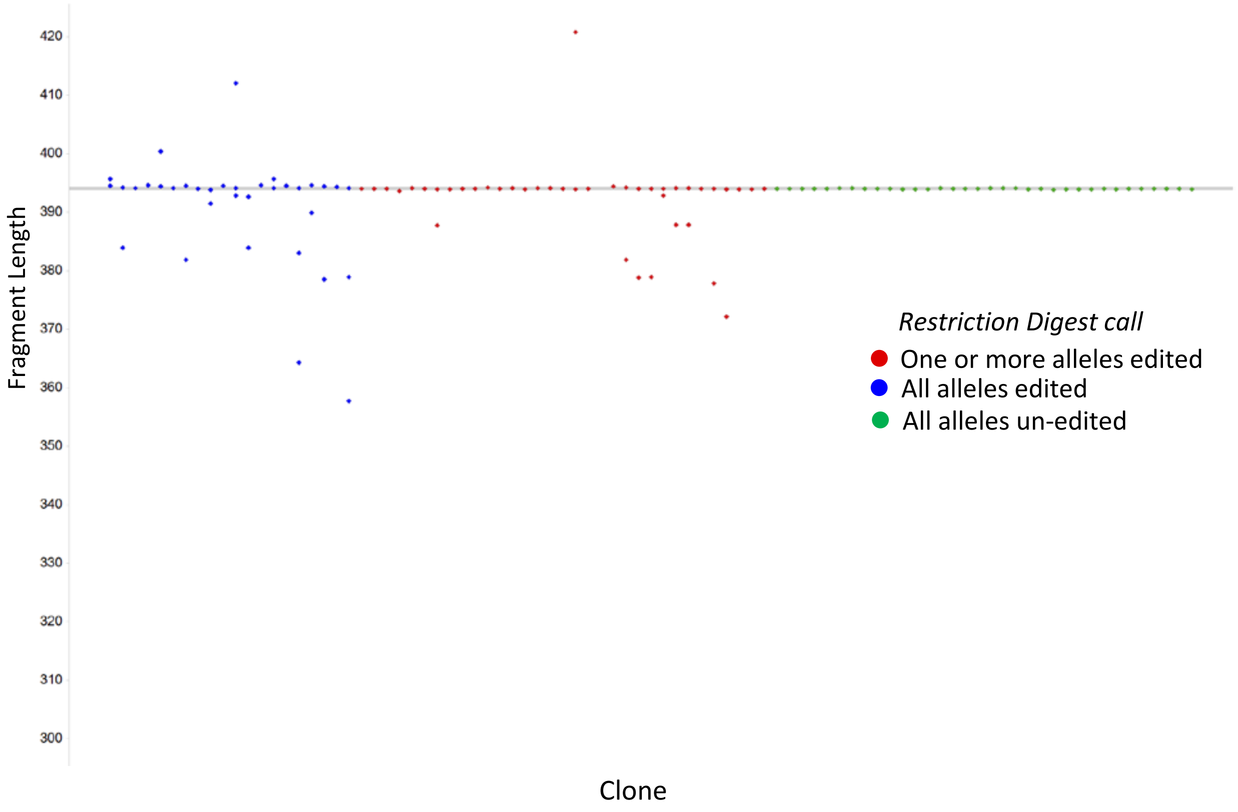

The common fragment lengths evident in these clones indicate that many of these clones are potentially sisters that arose from a small population of cells in which one allele of *EZH2* was initially edited. There are also a few clones in which there are 3 or more fragment length traces. Checking of the Cell Metric data indicate that these clones were not true ‘singles’ in that more than one cell was seeded in that particular well during the single cell cloning step. The grey line represents the wild-type fragment length (394 bp).

References

1. Chesworth, R., DUNCAN, K.W., Kawano, S., KEILHACK, H., KLAUS, C., Kuntz, K.W., Seki, M., SHIROTORI, S., WARHOLIC, N. and Zheng, W. (2012). WO2012142504 A1.
